# Stromal Down-Regulation of Macrophage CD4/CCR5 Expression and NF-κB Activation Mediates HIV-1 Non-Permissiveness in Intestinal Macrophages

**DOI:** 10.1371/journal.ppat.1002060

**Published:** 2011-05-26

**Authors:** Ruizhong Shen, Gang Meng, Christina Ochsenbauer, Paul R. Clapham, Jayleen Grams, Lea Novak, John C. Kappes, Lesley E. Smythies, Phillip D. Smith

**Affiliations:** 1 Department of Medicine (Gastroenterology), University of Alabama at Birmingham, Birmingham, Alabama, United Sates of America; 2 Lutheran Family Health Centers, Brooklyn, New York, United States of America; 3 Department of Medicine (Hematology/Oncology), University of Alabama at Birmingham, Birmingham, Alabama, United States of America; 4 Program in Molecular Medicine and Microbiology, University of Massachusetts, Worcester, Massachusetts, United States of America; 5 Department of Surgery (Gastrointestinal), University of Alabama at Birmingham, Birmingham, Alabama, United States of America; 6 Department of Pathology, University of Alabama at Birmingham, Birmingham, Alabama, United States of America; 7 VA Medical Center, Birmingham, Alabama, United States of America; NIH/NIAID, United States of America

## Abstract

Tissue macrophages are derived exclusively from blood monocytes, which as monocyte-derived macrophages support HIV-1 replication. However, among human tissue macrophages only intestinal macrophages are non-permissive to HIV-1, suggesting that the unique microenvironment in human intestinal mucosa renders lamina propria macrophages non-permissive to HIV-1. We investigated this hypothesis using blood monocytes and intestinal extracellular matrix (stroma)-conditioned media (S-CM) to model the exposure of newly recruited monocytes and resident macrophages to lamina propria stroma, where the cells take up residence in the intestinal mucosa. Exposure of monocytes to S-CM blocked up-regulation of CD4 and CCR5 expression during monocyte differentiation into macrophages and inhibited productive HIV-1 infection in differentiated macrophages. Importantly, exposure of monocyte-derived macrophages simultaneously to S-CM and HIV-1 also inhibited viral replication, and sorted CD4^+^ intestinal macrophages, a proportion of which expressed CCR5^+^, did not support HIV-1 replication, indicating that the non-permissiveness to HIV-1 was not due to reduced receptor expression alone. Consistent with this conclusion, S-CM also potently inhibited replication of HIV-1 pseudotyped with vesicular stomatitis virus glycoprotein, which provides CD4/CCR5-independent entry. Neutralization of TGF-β in S-CM and recombinant TGF-β studies showed that stromal TGF-β inhibited macrophage nuclear translocation of NF-κB and HIV-1 replication. Thus, the profound inability of intestinal macrophages to support productive HIV-1 infection is likely the consequence of microenvironmental down-regulation of macrophage HIV-1 receptor/coreceptor expression and NF-κB activation.

## Author Summary

Human intestinal macrophages, unlike lymphoid tissue macrophages, brain microglia and genital (vaginal) macrophages, are profoundly incapable of supporting productive HIV-1 infection. Intriguingly, all macrophages are derived exclusively from blood monocytes, which are HIV-1 permissive after differentiation into monocyte-derived macrophages. Therefore, the unique non-permissiveness of intestinal macrophages to HIV-1 must be conferred by the intestinal mucosal microenvironment. Here we report that intestinal stroma potently blocked up-regulation of HIV-1 receptor/coreceptor CD4 and CCR5 expression during monocyte differentiation into macrophages and macrophage nuclear translocation of NF-κB, which is a critical requirement for HIV-1 transcription. These two mechanisms work collaboratively to render intestinal macrophages non-permissive to HIV-1. Harnessing this natural antiviral defense may provide a novel strategy to exploit for the prevention of infection in HIV-1 permissive cells.

## Introduction

Macrophages play crucial roles in the establishment, pathogenesis and latency of human immunodeficiency virus-1 (HIV-1) infection [1], [2], [3] through their ability to support viral replication [4], [5], transmit virus [6] and act as a viral reservoir [6], [7], [8], [9]. In this connection, macrophages throughout the body, including lymphoid tissue macrophages [10], [11], brain microglia [12] and genital (vaginal) macrophages [13], are permissive to HIV-1. In sharp contrast, resident macrophages in the human small intestine are profoundly incapable of supporting productive HIV-1 infection [13], [14], [15], although intestinal macrophages are derived exclusively from blood monocytes [16], which when differentiated into monocyte-derived macrophages are HIV-1 permissive [4], [5], [17], [18]. The unique non-permissiveness of intestinal macrophages to HIV-1 stands in marked contrast to the ability of intestinal CD4^+^ T cells to support productive viral infection and undergo early, rapid and profound depletion during primary HIV-1 and SIV infection [19], [20], [21], [22], [23], [24], [25], [26].

After their recruitment into the lamina propria, pro-inflammatory blood monocytes differentiate into non-inflammatory intestinal macrophages through stromal transforming growth factor β (TGF-β)-mediated Smad-induced IκBα and nuclear factor kappa B (NF-κB) inactivation, as we recently reported [27], [28]. In further contrast to blood monocytes, intestinal macrophages are markedly down-regulated for receptors that mediate inflammatory responses, including LPS, Fcγ and Fcα receptors [27], [28], [29], triggering receptor expressed on myeloid cells-1 (TREM-1) [30], [31], as well as CD4, CCR5 and CXCR4 [13], [14], [15]. Since CCR5 expression correlates directly with the differentiation of monocytes into macrophages [32], [33], [34], the reduced expression of CCR5 on intestinal macrophages raises the possibility that the non-permissiveness of intestinal macrophages to HIV-1 is related to reduced HIV-1 receptor/co-receptor expression. However, our detection of proviral DNA in isolated intestinal macrophages exposed to HIV-1 *in vitro* [14] suggests post-entry restriction also may be involved in the inability of intestinal macrophages to support HIV-1 replication.

To elucidate the mechanism that renders intestinal macrophages non-permissive to HIV-1, we exposed blood monocytes and monocyte-derived macrophages to conditioned media from cultured lamina propria stroma isolated from normal human jejunum to determine the effect of the lamina propria microenvironment on CD4/CCR5 expression and HIV-1 permissiveness. Our results indicate that the inability of primary human intestinal macrophages to support HIV-1 replication is likely due not only to the marked down-regulation of CD4 and CCR5 but also to the inability of intestinal macrophages to activate NF-κB, a critical requirement for HIV-1 transcription.

## Results

### Intestinal macrophages express markedly reduced levels of CD4, CCR5 and CXCR4

CCR5-tropic HIV-1 strains are predominant among the transmitted/founder viruses isolated from acutely infected persons [35], [36], [37]. Since the gastrointestinal mucosa is the largest reservoir of macrophages in the body [38], and macrophages are an important HIV-1 target cell, we initiated studies to define the HIV-1 receptor phenotype and permissiveness of purified intestinal macrophages to macrophage-tropic HIV-1. Intestinal macrophages and blood monocytes were isolated from the same donors, purified and analyzed for expression of the HIV-1 primary receptor CD4 and the coreceptors CCR5 and CXCR4. As shown in **Table 1**, very low proportions of intestinal macrophages expressed CD4 (1.0%), CCR5 (0.8%) and CXCR4 (2.1%), and a barely detectable proportion (0.3%) expressed both CD4 and CCR5 (*P* = 0.0001 to *P* = 0.039), consistent with our earlier finding of markedly diminished CD4, CCR5 and CXCR4 expression on intestinal but not vaginal macrophages [13]. The low levels of CD4 and CCR5 expressed on intestinal macrophages corresponded to low levels of receptor/co-receptor-specific mRNA [13]. In contrast, modest proportions of blood monocytes expressed CD4 (11.6%), CCR5 (2.9%) and CXCR4 (14.1%), and 2.2% of the monocytes were CD4^+^CCR5^+^, indicating that 3- to 10-fold fewer intestinal macrophages expressed the receptors compared to autologous blood monocytes (**Table 1**).

**Table 1:** HIV-1 receptor and co-receptor expression on purified intestinal macrophages and autologous blood monocytes.

	Intestinal Macrophages		Monocytes		*P* value^c^
	Mean^a^	±SEM^b^	Mean	±SEM	
CD4^+^	1.0	0.3	11.6	2.0	0.0001
CCR5^+^	0.8	0.3	2.9	1.0	0.039
CXCR4^+^	2.1	0.7	14.1	4.4	0.008
CD4^+^CCR5^+^	0.3	0.1	2.2	0.6	0.001
CD4^+^CXCR4^+^	0.4	0.2	4.4	0.9	0.0002

a Mean percentage of positive cells (5 donors);

b SEM, standard error of mean;

c P value is calculated by Student t-test.

### CD4^+^ intestinal macrophages do not support HIV-1 replication

**Figure 1:**
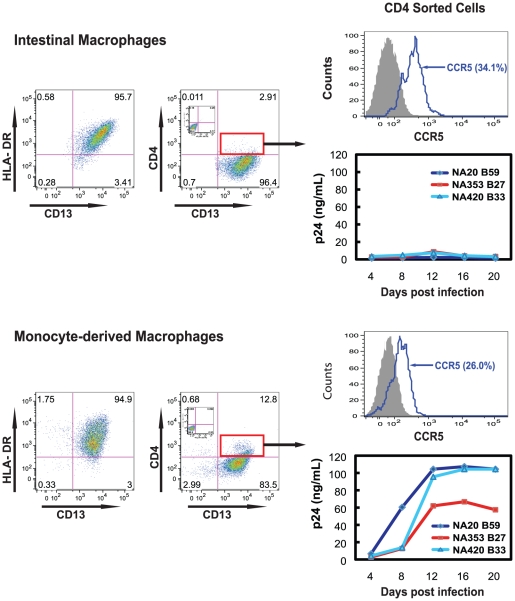
CD4^+^ intestinal macrophages do not support HIV-1 replication. CD4^+^ intestinal macrophages and blood monocytes were purified from jejunal and blood MNLs from the same donor by MACS sorting, cultured for 4 days, inoculated with highly macrophage-tropic R5 viruses (MOI = 1) and monitored for p24 production at 4-day intervals for 20 days (n = 2 donors, p24 determinations for each donor in triplicate). Inset dot plots show staining with isotype control antibodies. The % in the histograms indicates percentage of CCR5^+^ cells among CD13^+^CD4^+^ cells.

We previously showed that isolated intestinal macrophages do not support HIV-1 replication [13], [14], [15]. The low level of CD4, as well as CCR5, on intestinal macrophages (**Table 1**) raised the possibility that a restriction in HIV-1 entry could contribute to the cells' non-permissiveness to HIV-1. To address this possibility, we sorted autologous CD4^+^ intestinal macrophages and blood monocytes by magnetic activated cell sorting (MACS), cultured the cells for 4 days (>98% viable), inoculated each population with equivalent amounts of highly fusigenic and macrophage-tropic R5 viruses, including NA420 B33, NA20 B59 or NA353 B27, which infect cells with extremely low levels of CD4 and/or CCR5 expression [39], and monitored viral replication by p24 release over 20 days. As shown in **Figure 1**, 95% of both the intestinal macrophages and blood monocytes were HLA-DR^+^CD13^+^. Among the sorted CD4^+^ intestinal macrophages, 34.1% expressed CCR5 and levels of p24 were barely detectable only on day 12, whereas among the sorted CD4^+^ blood monocytes, 26% expressed CCR5 and large amounts of p24 were released by the monocyte-derived macrophages up to day 20 (**Figure 1**). Importantly, neither the exposure of intestinal macrophages to pro-inflammatory stimuli, including lipopolysacharride, interferon-γ or tumor necrosis factor-α, nor culture for up to 2 weeks prior to inoculation with virus, induced HIV-1 permissiveness in the macrophages (data not shown). These findings indicate that even CD4^+^ intestinal macrophages that express CCR5 are refractory to HIV-1, implicating a post-entry mechanism for down-regulated HIV-1 permissiveness. However, the profound low level of CD4 and CCR5 expression on the total intestinal macrophage population (**Table 1**) raised the possibility that the mucosal microenvironment of the jejunum caused the down-regulation of CD4 and CCR5, thereby also contributing to the reduced permissiveness of intestinal macrophages to CCR5-tropic HIV-1.

### Intestinal stroma-conditioned media (S-CM) blocks macrophage CD4 and CCR5 expression and HIV-1 replication

**Figure 2:**
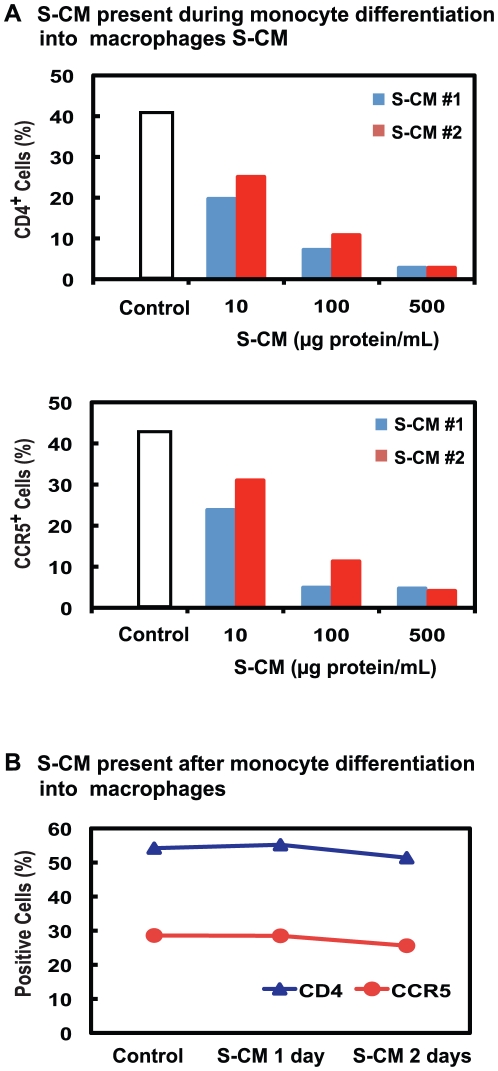
Intestinal S-CM down-regulates monocyte-derived macrophage CD4 and CCR5 expression during, but not after, differentiation. **(A)** Monocyte-derived macrophages were generated by culturing monocytes for 2 days in media alone (control) or media plus increasing concentrations of stoma-conditioned media (S-CM) derived from normal jejunum from 2 different tissue donors and then analyzed for CD4 and CCR5 expression by flow cytometry. Data are from a representative experiment from 4 blood monocyte donors. **(B)** Monocyte-derived macrophages were generated by culturing monocytes for 4 days in media alone (control), after which S-CM (100 µg protein/mL) was added for an additional 2 days of culture, and the cells were analyzed for CD4 and CCR5 expression. Values are the % cells positive for the indicated receptor in a representative experiment from 3 blood monocyte donors.

Intestinal macrophages are terminally differentiated and express very low levels of CD4 and CCR5, but they are derived from blood monocytes [16], which, during and after differentiation into adherent macrophages, express high levels of CD4 and CCR5. Since factors released by the intestinal extracellular matrix (stroma) down-regulate an array of innate response receptors on blood monocytes [27], we examined whether stromal factors present in conditioned media derived from normal intestinal stroma (S-CM) [27], [28] also down-regulate CD4 and CCR5 expression on blood monocytes during and after their differentiation into macrophages. Compared to monocytes differentiated into adherent macrophages during 2 days culture in media alone, monocytes differentiated into macrophages in the presence of S-CM (10–500 µg protein/mL) displayed a marked dose-dependent decrease in surface CD4 and CCR5 (**Figure 2A**). In contrast, when monocytes were first differentiated for 4 days into adherent macrophages and then exposed for 2 days to S-CM, CD4 and CCR5 expression was not down-regulated (**Figure 2B**). Thus, intestinal stromal products prevent differentiation-induced upregulation of CD4 and CCR5 expression on monocyte-derived macrophages but do not down-regulate receptor/co-receptor expression after the cells have differentiated into macrophages. These findings offer an explanation for the near absence of CD4 and CCR5 on terminally differentiated intestinal macrophages, which are derived exclusively from circulating monocytes that have recruited into the lamina propria.

**Figure 3:**
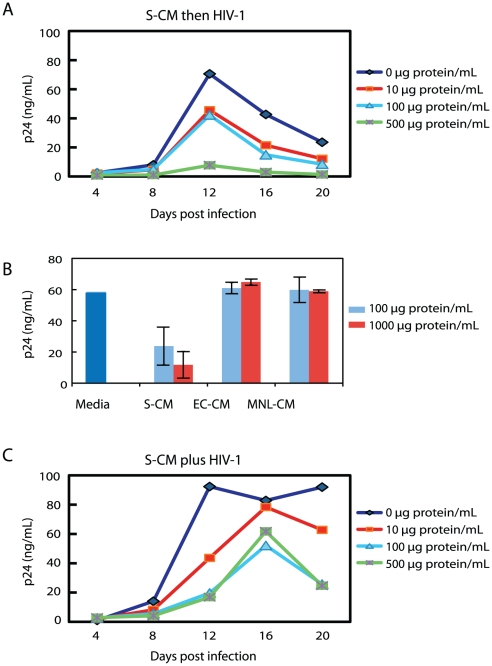
Intestinal S-CM down-regulates HIV-1 replication in monocyte-derived macrophages. **(A)** MACS-sorted monocytes were cultured for 4 days in M-CSF and the resultant monocyte-derived macrophages were cultured for an additional 2 days in the presence of S-CM at the indicated concentrations, then inoculated with R5 virus (NA353 B27; MOI = 1) for 2 hours and monitored for p24 release at 4-day intervals for 20 days (n = 4 donors, each in triplicate). **(B)** Monocyte-derived macrophages were cultured for 2 days with intestinal epithelial cell-conditioned media (EC-CM), mononuclear leukocyte (MNL)-CM or S-CM derived from the same normal jejunal tissue at the indicated concentrations, inoculated with R5 virus in triplicate as above, and analyzed for p24 release on day 12 (mean ± SD; n = 3). **(C)** Monocyte-derived macrophages prepared as in **A** were inoculated simultaneously with R5 virus (NA353 B27; MOI = 1) and S-CM, cultured for 2 hrs and then monitored for p24 release as in **A** (n = 4 donors, each in triplicate).

Since undifferentiated monocytes do not support productive HIV-1 infection, we next determined whether monocyte-derived macrophages exposed to lamina propria stromal products supported HIV-1 replication. Monocyte-derived macrophages were cultured for 2 days in the presence of varying concentrations of S-CM, after which the cultures were inoculated with R5 virus (NA353 B27). As shown in **Figure 3A**, the pre-incubation of monocyte-derived macrophages with S-CM prior to the inoculation of HIV-1 caused a dose-dependent decrease in p24 production during a 20-day culture period. However, when monocyte-derived macrophages were pre-incubated with conditioned media from purified cultures of intestinal epithelial cells (EC-CM) [40] or intestinal mononuclear cells (MNL-CM) [27] derived from the same donor tissue as the S-CM, HIV-1 replication was not inhibited (**Figure 3B**). Furthermore, S-CM also caused a dose-dependent decrease in viral replication when S-CM and virus were added simultaneously to the monocyte-derived macrophage cultures (**Figure 3C**). These findings suggest that extracellular matrix products, rather than intestinal epithelial cell or lamina propria mononuclear cell products, inhibit productive HIV-1 infection in intestinal macrophages and that the down-regulation in viral replication is not the exclusive consequence of the low level of CD4 and CCR5 expression on the macrophages.

### S-CM blocks macrophage permissiveness to vesicular stomatitis virus glycoprotein (VSV-G) pseudotyped virus

**Figure 4:**
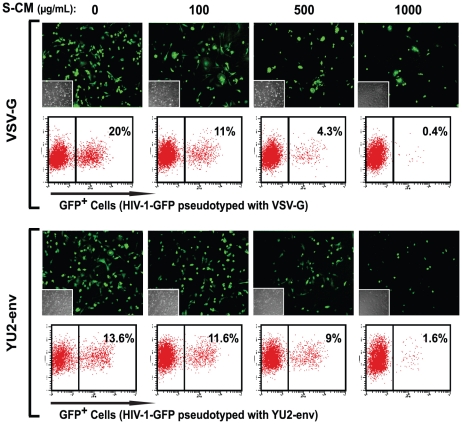
Intestinal S-CM blocks replication of VSV-G and YU2 env pseudotyped virus. Monocytes were cultured for 4 days, inoculated with VSV-G or YU2 env pseudotyped GFP reporter virus and cultured for an additional 2 days, after which GFP expression was analyzed by fluorescence microscopy and flow cytometry in a representative experiment using monocyte-derived macrophages and S-CM from separate donors (n = 3).

To further distinguish between reduced HIV-1 entry and down-regulated viral replication, we pseudotyped HIV-1 with VSV-G envelope to bypass HIV-1 receptor/co-receptor-dependent entry. As predicted, treatment of monocyte-derived macrophages with S-CM for up to 24 hours did not impair the entry of VSV-G pseudotyped virus into the cells (data not shown) but caused a dose-dependent reduction in single-round replication of VSV-G pseudovirons, as shown by immunofluorescence and flow cytometry in **Figure 4**, **upper panels**. The same pre-treatment of monocyte-derived macrophages with S-CM also inhibited infection of YU2 pseudovirons in a dose-dependent manner (**Figure 4**, **lower panels**). These results further indicate that S-CM inhibition of R5 replication was not due only to down-regulated CD4 and CCR5 expression but also involved post-entry restriction in viral replication.

### Stromal TGF-β inhibits NF-κB activation and down-regulates HIV-1 replication in monocyte-derived macrophages

**Figure 5:**
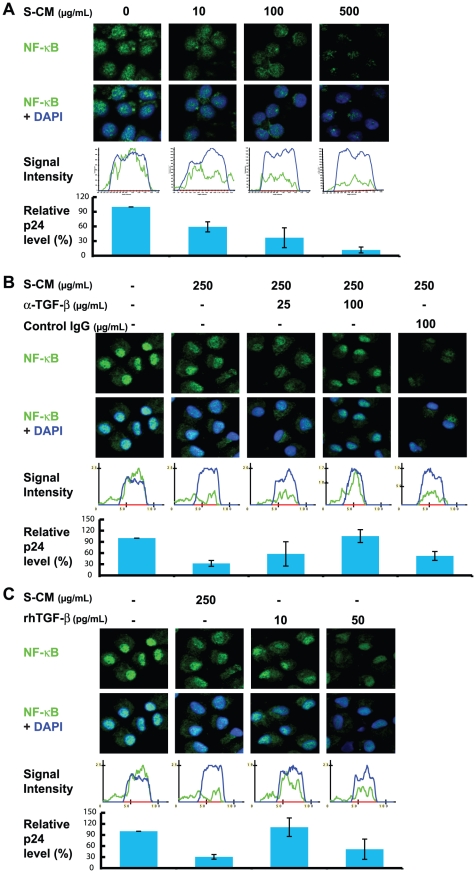
Down-regulation of NF-κB by S-CM correlates with down-regulation in the cells' ability to support HIV-1 replication. (**A**) Monocytes were cultured in media plus M-CSF and then inoculated in triplicate with R5 HIV-1 (NA353 B27; MOI = 1) plus S-CM at the indicated concentration for 2 hours. Cells were evaluated for NF-κB p65 translocation by confocal microscopy and NF-κB intensity by IPLab image analysis software after 2 hours and for viral replication by p24 ELISA on day 12 (n = 3 donors). Histograms are representative of a single experiment and show distribution of NF-κB (green line) in relation to the nucleus (blue line). The p24 value of each treatment was normalized to the media control group with the replication level of media control group defined as 100%. Data shown are the means of relative p24 levels from independent experiments with 3 donors. (**B**) Anti-TGF-β antibodies reverse the inactivation of NF-κB and S-CM-mediated down-regulation of HIV-1 replication. Experiments were performed as in **A** except the S-CM (250 µg protein/mL) was pre-incubated for 1 hour with anti-TGF-β antibodies at the indicated concentration (n = 3 donors). (**C**) Recombinant human TGF-β reduces NF-κB translocation and R5 virus replication. Experiments were performed as in **A**, except the S-CM was replaced with rhTGF-β at 10 or 50 pg/mL (n = 4 donors).

We have shown that stromal TGF-β inactivates NF-κB in monocyte-derived macrophages by dysregulating NF-κB signal proteins and inducing IκBα, the cytoplasmic negative regulator of NF-κB [28]. Because NF-κB is required for HIV-1 transcription [41], we investigated whether stromal TGF-β-mediated down-regulation of NF-κB also inhibits the ability of monocyte-derived macrophages to support HIV-1 replication. Monocyte-derived macrophages were cultured in triplicate with increasing concentrations of S-CM and inoculated with R5 HIV-1 (NA353 B27) at a multiplicity of infection (MOI) of 1. After 2 hours, cells were visualized by confocal microscopy for the translocation of phosphorylated NF-κB p65 (pNF-κB p65) into the nucleus and the cytoplasmic and nuclear intensity of NF-κB. On day 12, the supernatants in parallel cultures were analyzed for the level of p24. As shown in **Figure 5A**, exposure of monocyte-derived macrophages to increasing concentrations of S-CM caused a dose-dependent decrease in NF-κB p65 translocation into the nucleus and a dose-dependent decrease in p24 production. However, when S-CM at an inhibitory concentration of 250 µg protein/mL was pre-incubated for 1 hour with anti-TGF-β antibodies at a concentration of 100 µg/mL, S-CM inhibition of both the nuclear translocation of NF-κB p65 and HIV-1 p24 production was reversed, whereas pre-incubation with irrelevant IgG (100 µg/mL) antibody had no effect on S-CM inhibitory activities (**Figure 5B**). Furthermore, incubation of the cells with activated, recombinant human TGF-β (rhTGF-β at a concentration of 10 pg/mL had little or minimal effect on NF-κB translocation or p24 production (**Figure 5C**). However, rhTGF-β 50 pg/mL, which approximates the concentration of TGF-β in S-CM 250 µg/mL, inhibited NF-κB translocation and activity, as well as p24 production, similar to that of S-CM 250 µg/mL (**Figure 5C**). Moreover, we previously showed (flow cytometry, ELISA, immunocytochemistry and Western blot) that LPS-exposed intestinal macrophages and S-CM-treated blood monocytes did not phosphorylate p65, had very low levels of p50, did not translocate p50 or p65 into the nucleus and expressed markedly reduced levels of NF-κB signal proteins (28). Expression of p50 and p65 genes also were markedly reduced in intestinal macrophages compared to autologous blood monocytes (28). These findings are consistent with minimal, if any, transcriptionally active p50/p65 heterodimer and together implicate stromal TGF-β-mediated down-regulation of NF-κB activation in the inhibition of HIV-1 replication by stromal factor-differentiated macrophages *in vitro* and intestinal macrophages *in vivo*.

## Discussion

We have shown that macrophages isolated from normal human small intestine are highly refractory to productive HIV-1 infection [13], [14], [15], supporting observations that memory CD4^+^ T cells rather than macrophages are the predominant mononuclear target cell in the intestinal mucosa during primary HIV-1 infection [19], [20], [21], [22], [23], [24], [25], [26]. We also have shown that in contrast to intestinal macrophages, vaginal macrophages are permissive to macrophage-tropic HIV-1 [13]. Since tissue macrophages throughout the body are derived from blood monocytes, our findings suggest that the lamina propria of the intestinal mucosa is a unique microenvironment capable of influencing HIV-1 permissiveness in blood monocytes recruited to the intestinal mucosa. Consistent with this concept, we present new evidence that products released by the intestinal extracellular matrix inhibit up-regulation of CD4 and CCR5 during the differentiation of blood monocytes into macrophages. However, the low level of CD4 and CCR5 expression on intestinal macrophages is not the exclusive cause of the cells' non-permissiveness to HIV-1, since (1) the very small subset (1%) of intestinal macrophages that express CD4, a proportion of which also express CCR5, did not support HIV-1 replication; (2) intestinal stromal products also decreased HIV-1 replication when stromal products were added simultaneously to cultures of monocyte-derived macrophages, i.e., before the induction of CD4 and CCR5 down-regulation; and (3) stromal products inhibited single-round gene expression of VSV-G pseudotyped virus, which enters cells independent of CD4 and CCR5. In this connection, we previously showed that unsorted intestinal macrophages with undetectable CD4 also do not of support HIV-1 replication (13, 14). Having previously shown that stromal TGF-β differentiates pro-inflammatory blood monocytes into non-inflammatory cells with the phenotype and function of intestinal macrophages [27] through Smad-induced IκBα expression and NF-κB signal dysregulation [28], we show here that a critical consequence of stromal TGF-β-induced NF-κB inactivation is the profound inability of monocyte-derived macrophages to support HIV-1 replication.

TGF-β is reported to both inhibit and stimulate HIV-1 replication, depending on the cell type, level of cell differentiation, virus strain, timing of treatment and presence of other cytokines [42], [43], [44]. In intestinal mucosa, latent TGF-β is produced by many different types of cells, including epithelial cells, mast cells, T regulatory cells, T cells undergoing apoptosis, and stromal cells. TGF-β constitutively released by these cells binds to the lamina propria extracellular matrix binding domains and upon activation and release regulates multiple macrophage defense and immune functions, consistent with an elaborate and finely tuned system of cross-talk that we have described previously [16]. Here we show that among these functions is the down-regulation of NF-κB activity and thus HIV-1 replication in monocyte-derived macrophages. These data suggest that TGF-β, at least in part, mediates the profound non-permissiveness of intestinal macrophages to HIV-1.

NF-κB plays a critical role in HIV-1 replication in T cells [41] and cells of the monocyte lineage [45]. In addition to stimulating the initiation of HIV-1 transcription [46], [47], [48], NF-κB also has been implicated in promoting HIV-1 transcriptional elongation [49], [50]. Importantly, NF-κB is constitutively activated in HIV-1-infected monocytes [51], possibly through upstream activation of the IKK complex by HIV-1 regulatory/accessory proteins [52], [53] or HIV-1-induced (via NF-κB activation) cytokines [54]. The activation of IKK leads to the phosphorylation and proteosomal degradation of IκBα and IκBβ, thereby releasing NF-κB for translocation into the nucleus to bind NF-κB-binding sites in the enhancer region of the HIV-1 long terminal repeat and host gene promotor sites. Thus, we conclude that stromal TGF-β inactivates NF-κB in monocyte-derived macrophages and that this inactivation likely contributes to the profound blockade in HIV-1 expression in intestinal macrophages, a highly unique population of mononuclear phagocytes [55], [56].

The HIV-1 non-permissiveness of intestinal macrophages due to NF-κB inactivation is consistent with our recent finding that stromal TGF-β dysregulation of NF-κB signaling causes inflammation anergy in intestinal macrophages [28]. Importantly, long-term culture of intestinal macrophages in the absence of stromal factors does not restore inflammatory capability [27], [28] and, as reported here, did not promote the emergence of HIV-1 permissiveness, indicating prolonged, if not permanent, down-regulation of these functions in intestinal macrophages. Also, exposure of intestinal macrophages to pro-inflammatory stimuli, including lipopolysaccharide (LPS), interferon-γ (IFN-γ) and tumor necrosis factor-α (TNF-α), does not induce inflammatory function [27], [28] and did not restore replication competence. These findings suggest that in primary HIV-1 infection, resident macrophages in healthy intestinal mucosa are incapable of *de novo* HIV-1 replication.

In contrast to primary HIV-1 infection, in late stage disease HIV-1-infected blood monocytes may recruit to intestinal mucosa that is either inflamed or infected with opportunistic pathogens. In such a microenvironment, dysregulated homeostasis permits viral replication to continue after the monocytes take up residence in the lamina propria, as we have reported for esophageal macrophages in patients with AIDS and opportunistic mucosal infections [57]. We also have reported that cytomegalovirus blocks stromal inhibition of HIV-1 infection of macrophages and that this inhibition is mediated, at least in part, by cytomegalovirus-induced monocyte production of TNF-α, which acts *in trans* to enhance HIV-1 replication [58]. However, the very low levels of TNF-α (<2.9 pg/mL) in S-CM generated from normal mucosa and inflamed Crohn's mucosa [59] suggest that TNF-α is not involved in stromal down-regulation of intestinal macrophage permissiveness to HIV-1.

In the present study, we investigated HIV-1 permissiveness in intestinal macrophages using highly macrophage-tropic R5 viruses, including NA420 B33, NA20 B59 and NA353 B27 [39], [59], in order to maximize the possibility of infecting intestinal macrophages. Interestingly, infectious molecular clones of transmitted founder viruses derived from acutely infected persons are R5-tropic but fail to replicate efficiently in monocyte-derived macrophages [60], [61]. Although we have not yet examined the ability of these molecular clones to infect intestinal macrophages, such infection seems unlikely, since intestinal macrophages do not activate NF-κB, a requirement for HIV-1 gene transcription during macrophage differentiation [45].

The findings presented here do not exclude the possibility that HIV-1 restriction factors other than TGF-β are present in the stroma and thus S-CM. S-CM was used in a range of 10-1000 µg protein/mL, corresponding to TGF-β in the range of 1–150 pg/mL. Although rhTGF-β at a concentration of 10 pg/L had little or minimal effect on NF-κB translocation or p24 production (Figure 5C), rhTGF-β 50 µg/mL, which approximates the concentration of TGF-β in S-CM 250 µg/mL, inhibited NF-κB translocation and viral replication (p24 production), similar to that of S-CM 250 µg/mL. Apolipoprotein B mRNA-editing enzyme catalytic polypeptide-like 3G (APOBEC3G), which causes dC-to-dU mutations in viral DNA, is reported to be induced by LPS in dendritic cells and by IFN-α in monocyte-derived macrophages [62], [63]; however, we have been unable to detect APOBEC3G in resting or IFN-α-treated intestinal macrophages. Also, higher levels of anti-HIV-1 miRNAs have been reported to inhibit HIV-1 in monocytes [64], [65], but the role of miRNA as a restriction factor in monocytes is controversial [66], [67]. A cellular restriction factor that is neutralized by primate lentiviral Vpx protein was recently detected in quiescent monocytes, but its reduction as the cells differentiate into macrophages makes it an unlikely restriction factor in terminally differentiated intestinal macrophages [68]. Other potential restriction factors, including p21 [69], [70] and interferon-induced C/EBPβ [71], [72], have been proposed but have not yet been investigated in mucosal macrophages.

A confounding issue regarding post-entry restrictions in intestinal macrophages is that such restrictions would be unique to macrophages residing in the intestinal mucosa, since macrophages in the vaginal mucosa are highly replication competent [13]. Although the extracellular matrix could release products that induce yet-to-be-identified anti-viral restrictions, the findings presented here implicate stromal TGF-β-induced NF-κB inactivation as contributing to the non-permissiveness of macrophages in the human small intestine. These findings help explain the overwhelming absence of productive infection in intestinal macrophages, in sharp contrast to the highly productive infection in intestinal T cells, in primary HIV-1 infection. The ability of intestinal CD4^+^ T cells to support robust HIV-1 replication is well established in our *in vitro* [13] and *in vivo* studies [9], [10], [11], [12], [13], [14], [15], [16], [17], [18], [19], [20], [21], [22], [23], [24], [25], [26]. Furthermore, TGF-β does not inhibit HIV-1 expression in a chronically infected T cell line or in primary T cell blasts infected *in vitro* with HIV-1 [42]. The discordance between intestinal T cell and macrophage support for HIV-1 replication in the presence of down-regulatory stromal TGF-β is currently under investigation in our laboratory. Thus, the unique dysregulation in NF-κB signaling induced in monocytes by extracellular matrix products, especially TGF-β, when the cells take up residence in the intestinal mucosa, offers a mechanism by which the host down-regulates mucosal macrophages for harmful pro-inflammatory responses and permissiveness to viruses in which transcription is NF-κB-dependent. Harnessing this natural anti-viral defense mechanism may provide a novel strategy to exploit for the prevention of infection in HIV-1 permissive cells.

## Materials and Methods

### Ethics statement

All tissue and cell protocols were approved by the Institutional Review Board of the University of Alabama at Birmingham. Written informed consent was provided by study participants.

### Intestinal macrophages and blood monocytes

Macrophages were isolated from segments of intestinal mucosa of otherwise healthy subjects undergoing elective gastric bypass by enzyme digestion and purified by counterflow centrifugal elutriation, as previously described [73], [74], [75]. Circulating blood monocytes from the same donors were purified by gradient sedimentation followed by magnetic anti-PE bead isolation of anti-CD14-PE-treated cells per the manufacture's manual (Miltenyi Biotec). All studies were performed using fresh cells. Macrophages and monocytes were routinely >98% pure and 98% viable by propidium iodide staining. CD4^+^ monocytes and intestinal macrophages were isolated by magnetic CD4^+^ microbead separation.

### HIV-1 molecular clones and viruses

Macrophage-tropic viruses were prepared as previously described [13], [76], [77]. Briefly, replication competent clones of highly macrophage-tropic R5 viruses, including NA420 B33, NA20 B59 and NA353 B27 [39], were transfected into 293T cells by Fugene 6 (Roche), according to the manufacture's protocol. After 60 hours, the supernatants were harvested, clarified by low speed centrifugation (1,000 *g*, 10 minutes), filtered (0.45 µm filter), titrated using JC53BL cells [78], aliquoted and stored at −80°C.

YU2 envelope (Env) or vesicular stomatitis virus glycoprotein (VSV-G) HIV-1 pseudovirions that express GFP upon infection were kindly provided by D. Levy, NYU and constructed as follows. Briefly, the *env* gene was deleted and the *gfp* gene was inserted between the *env* and *nef* genes of the pNL4-3 clone. An internal ribosome entry site (IRES) element was inserted between the *gfp* and *nef* genes to rescue *nef* gene expression [79]. To generate the YU2 Env or VSV-G GFP reporter pseudovirions, the clone was co-transfected with the YU2 Env or VSV-G expression plasmid into 293T cells and harvested, as described above.

### Conditioned media

Using our previously described protocols [40], [73], [74], epithelium and lamina propria mononuclear cells (MNLs) were removed by enzyme digestion from segments of normal human jejunum from otherwise healthy subjects undergoing elective gastric bypass, and purified by elutriation. The epithelial cells (EC) (10×10^6^/mL), lamina propria MNLs (10×10^6^/mL), and cell-depleted lamina propria stroma (1 g wet wt stromal tissue/mL), respectively, were cultured in RPMI for 24 hours without serum, and the EC-conditioned media (EC-CM), MNL-CM and stroma-CM (S-CM) were harvested, sterile-filtered (0.2 mm Syringe Filter; Corning Inc.) and frozen at −70°C, as previously described [27], [28]. Cell depletion from lamina propria stroma was confirmed by immunohistochemistry [73]; intestinal macrophages expressed barely detectable CD14 [13]. Conditioned media did not alter monocyte-derived macrophage viability during incubation for as long as 4 days as assessed by flow cytometric analysis of propidium iodide uptake. S-CMs were normalized to 500 µg/mL RPMI. Endotoxin and protein content were determined by ELISA (endotoxin ELISA: Cambrex Bio Science; protein ELISA: Pierce Protein Research Products/Thermo Scientific). Only endotoxin-free EC-CM, MNL-CM and S-CM were used in the experiments.

### Flow cytometry

Intestinal macrophages and monocytes were incubated with optimal concentrations of PE-, APC-, or FITC-conjugated antibodies to HLA-DR, CD13, CD4, CCR5 (BD Pharmingen), or control mAbs of the same isotype at 4°C for 20 minutes, washed with PBS, fixed with 1% paraformaldehyde and analyzed by flow cytometry. Data were analyzed with FlowJo software (Tree Star, Inc.). To examine the effect of S-CM on CD4 and CCR5 expression in monocyte-derived macrophages, blood monocytes were cultured in 48-well plates at 5×10^5^ cells/well in RPMI plus macrophage colony-stimulating factor (M-CSF) serum and S-CM at final concentrations of 0, 10, 100 and 500 µg/mL for up to 3 days and analyzed for CD4 and CCR5. Student's *t*-test was used to determine the statistical significance of the difference of expression levels of these receptors between intestinal macrophages and autologous blood.

### HIV-1 infection of intestinal macrophages and monocyte-derived macrophages

Sorted intestinal macrophages and monocytes from 2 donors were cultured in triplicate in 96-well plates at 2×10^5^ cells/well in RPMI plus M-CSF and serum for 4 days. Cultures then were inoculated with NA20 B59, NA353 B27 or NA420 B33 at an MOI = 1, cultured for the indicated duration with 100 µL of supernatant, harvested every 4 days and stored at −70°C until assayed for p24 by ELISA (PerkinElmer).

To examine the effect of S-CM on macrophage permissiveness to HIV-1, MACS-sorted monocytes were cultured for 4 days in RPMI plus M-CSF to generate monocyte-derived macrophages, after which S-CM was added at final concentrations of 10, 100 and 500 µg protein/mL. Control cultures of monocyte-derived macrophages were incubated in media alone. Two days later, culture supernatants were removed, and triplicate cultures were inoculated with NA353 B27 (MOI = 1) for 2 hours, cultured for 20 days, and the kinetics of p24 production was determined as above. Parallel triplicate cultures of monocyte-derived macrophages were inoculated simultaneously with NA353 B27 (MOI = 1) plus S-CM (final concentrations of 0, 10, 100 and 500 µg protein/mL) for 2 hours, and viral replication was monitored as above.

Cultures of monocyte-derived macrophages prepared as above were inoculated with NA353 B27 (MOI = 1) plus S-CM or with S-CM only. Cells treated with S-CM only were harvested after 2 hours, cytospun onto glass slides and stained for NF-κB p65. Cells infected with virus were cultured, and supernatants were harvested on day 12 and assayed for p24 by ELISA. Parallel monocyte-derived macrophages were inoculated for 2 hours in triplicate with NA353 B27 (MOI = 1) plus S-CM 250 µg protein/mL pre-treated with 0, 25 or 100 µg/mL of anti- TGF-β for 1 hour at 37°C. Analysis of viral replication and NF-κB p65 staining were performed as above.

A final aliquot of monocyte-derived macrophages prepared as above was cultured for 6 days, inoculated in triplicate with NA353 B27 (MOI = 1) plus rhTGF-β (R&D Systems) or rhTGF-β only at final concentrations of 0, 10, or 50 pg/mL for 2 hours. Evaluation of NF-κB p65 intensity and viral replication were performed as above.

### Immunofluorescence staining for NF-κB p65

Cells cytospun onto glass slides were fixed and permeabilized with Cytofix/Cytoperm (BD Biosciences) for 20 minutes. After washing with PBS, cells were blocked with casein protein (DAKO) for 1 hour and incubated with rabbit anti-NF-κB p65 or isotype control antibodies (Santa Cruz Biotechnology) for 90 minutes, washed with PBS, incubated with donkey anti-rabbit IgG-FITC (Jackson ImmunoResearch Laboratories) for 30 minutes, washed with PBS and counterstained with DAPI nuclear stain. Cells were visualized by confocal microscopy, and the cytoplasmic and nuclear fluorescence intensity of NF-κB was converted to histograms using IPLab image analysis software version 3.6 (BD Biosciences Bioimaging).

For comparison of the effects of treatment on NK-κB activity, NF-κB intensity was normalized to the blue signal in the nucleus. Five images were analyzed per sample and mean intensities were generated. For comparison of the effects of treatment on HIV-1 replication, p24 value of each treatment was normalized to the media control group with the replication level of the media control group defined as 100%. Statistical significance was determined by Student's *t*-test.

### Statistical analysis

Data is expressed as mean ± SD or ± SEM, and statistical significance between groups was determined using Student's *t*-test. *P* values ≤0.05 were considered significant.

## References

[1] Kedzierska K, Crowe SM, Turville S, Cunningham AL (2003). The influence of cytokines, chemokines and their receptors on HIV-1 replication in monocytes and macrophages.. Rev Med Virol.

[2] Gorry PR, Churchill M, Crowe SM, Cunningham AL, Gabuzda D (2005). Pathogenesis of macrophage tropic HIV-1.. Curr HIV Res.

[3] Carter CA, Ehrlich LS (2008). Cell biology of HIV-1 infection of macrophages.. Annu Rev Microbiol.

[4] Collman R, Hassan NF, Walker R, Godfrey B, Cutilli J (1989). Infection of monocyte-derived macrophages with human immunodeficiency virus type 1 (HIV-1). Monocyte-tropic and lymphocyte-tropic strains of HIV-1 show distinctive patterns of replication in a panel of cell types.. J Exp Med.

[5] Rich EA, Chen IS, Zack JA, Leonard ML, O'Brien WA (1992). Increased susceptibility of differentiated mononuclear phagocytes to productive infection with human immunodeficiency virus-1 (HIV-1).. J Clin Invest.

[6] Sharova N, Swingler C, Sharkey M, Stevenson M (2005). Macrophages archive HIV-1 virions for dissemination *in trans*.. EMBO J.

[7] Embretson J, Zupancic M, Ribas JL, Burke A, Racz P (1993). Massive covert infection of helper T lymphocytes and macrophages by HIV during the incubation period of AIDS.. Nature.

[8] Zhu T, Muthui D, Holte S, Nickle D, Feng F (2002). Evidence for human immunodeficiency virus type 1 replication in vivo in CD14^+^ monocytes and its potential role as a source of virus in patients on highly active antiretroviral therapy.. J Virol.

[9] Crowe S, Zhu T, Muller WA (2003). The contribution of monocyte infection and trafficking to viral persistence, and maintenance of the viral reservoir in HIV infection.. J Leukoc Biol.

[10] Orenstein JM, Fox C, Wahl SM (1997). Macrophages as a source of HIV during opportunistic infections.. Science.

[11] Wahl SM, Greenwell-Wild T, Peng G, Hale-Donze H, Doherty TM (1998). *Mycobacterium avium* complex (MAC) augments macrophage HIV-1 production and increases CCR5 expression.. Proc Natl Acad Sci USA.

[12] Wahl SM, Allen JB, McCartney-Francis N, Morganti-Kossmann MC, Kossmann T (1991). Macrophage- and astrocyte-derived transforming growth factor β as a mediator of central nervous system dysfunction in acquired immune deficiency syndrome.. J Exp Med.

[13] Shen R, Richter HE, Clements RH, Novak L, Huff K (2009). Macrophages in vaginal but not in intestinal mucosa are monocyte-like and permissive to HIV-1.. J Virol.

[14] Li L, Meng G, Graham MF, Shaw GM, Smith PD (1999). Intestinal macrophages display reduced permissiveness to human immunodeficiency virus 1 and decreased surface CCR5.. Gastroenterology.

[15] Meng G, Sellers M, Mosteller-Barnum M, Rogers T, Shaw G (2000). Lamina propria lymphocytes, not macrophages, express CCR5 and CXCR4 and are the likely target cell for human immunodeficiency virus type 1 in the intestinal mucosa.. J Infect Dis.

[16] Smythies LE, Maheshwari A, Clements RH, Eckhoff D, Novak L (2006). Mucosal IL-8 and TGF-β recruit blood monocytes: Evidence for cross-talk between the lamina propria stroma and myeloid cells.. J Leukoc Biol.

[17] Sonza S, Maerz A, Deacon N, Meanger J, Mills J (1996). Human immunodeficiency virus type 1 replication is blocked prior to reverse transcription and integration in freshly isolated peripheral blood monocytes.. J Virol.

[18] Naif HM, Li S, Alali M, Chang J, Mayne C (1999). Definition of the stage of host cell genetic restriction of replication of human immunodeficiency virus type 1 in monocytes and monocyte-derived macrophages by using twins.. J Virol.

[19] Smit-McBride Z, Mattapallil JJ, McChesney M, Ferrick D, Dandekar S (1998). Gastrointestinal T lymphocytes retain high potential for cytokine responses but have severe CD4^+^ T-cell depletion at all stages of simian immunodeficiency virus infection compared to peripheral lymphocytes.. J Virol.

[20] Veazey RS, DeMaria M, Chalifoux LV, Shvetz DE, Pauley DR (1998). Gastrointestinal tract as a major site of CD4^+^ T cell depletion and viral replication in SIV infection.. Science.

[21] Guadalupe M, Reay E, Sankaran S, Prindiville T, Flamm J (2003). Severe CD4^+^ T cell depletion in gut lymphoid tissue during primary human immunodeficiency virus type 1 infection and substantial delay in restoration following highly active antiretroviral therapy.. J Virol.

[22] Brenchley JM, Hill BJ, Ambrozak DR, Price DA, Guenaga FJ (2004). T-cell subsets that harbor human immunodeficiency virus (HIV) *in vivo*: implications for HIV pathogenesis.. J Virol.

[23] Mehandru S, Poles MA, Tenner-Racz K, Horowitz A, Hurley A (2004). Primary HIV-1 infection is associated with preferential depletion of CD4^+^ T lymphocytes from effector sites in the gastrointestinal tract.. J Exp Med.

[24] Li Q, Duan L, Estes JD, Ma Z-M, Rourke T (2005). Peak SIV replication in resting memory CD4^+^ T cells depletes gut lamina propria CD4^+^ T cells.. Nature.

[25] Mattapallil JJ, Douek DC, Hill B, Nishimura Y, Martin M (2005). Massive infection and loss of memory CD4^+^ T cells in multiple tissues during acute SIV infection.. Nature.

[26] Mehandru S, Poles MA, Tenner-Racz K, Manuelli V, Jean-Pierre P (2007). Mechanisms of gastrointestinal CD4^+^ T-cell depletion during acute and early human immunodeficiency virus type 1 infection.. J Virol.

[27] Smythies LE, Sellers M, Clements RH, Mosteller-Barnum M, Meng G (2005). Human intestinal macrophages display profound inflammatory anergy despite avid phagocytic and bacteriocidal activity.. J Clin Invest.

[28] Smythies LE, Shen R, Bimczok D, Novak L, Clements RH (2010). Inflammation anergy in human intestinal macrophages is due to Smad-induced IκBα expression and NF-κB inactivation.. J Biol Chem.

[29] Smith PD, Smythies LE, Mosteller-Barnum M, Sibley DA, Russell MW (2001). Intestinal macrophages lack CD14 and CD89 and consequently are down-regulated for LPS- and IgA-mediated activities.. J Immunol.

[30] Schenk M, Bouchon A, Birrer S, Colonna M, Mueller C (2005). Macrophages expressing triggering receptor expressed on myeloid cells-1 are underrepresented in the human intestine.. J Immunol.

[31] Schenk M, Bouchon A, Seibold F, Mueller C (2007). TREM-1-expressing intestinal macrophages crucially amplify chronic inflammation in experimental colitis and inflammatory bowel diseases.. J Clin Invest.

[32] Rana S, Besson G, Cook DG, Rucker J, Smyth RJ (1997). Role of CCR5 in infection of primary macrophages and lymphocytes by macrophage-tropic strains of human immunodeficiency virus: resistance to patient-derived and prototype isolates resulting from the delta*ccr5* mutation.. J Virol.

[33] Naif HM, Li S, Alali M, Sloane A, Wu L (1998). CCR5 expression correlates with susceptibility of maturing monocytes to human immunodeficiency virus type 1 infection.. J Virol.

[34] Tuttle DL, Harrison JK, Anders C, Sleasman JW, Goodenow MM (1998). Expression of CCR5 increases during monocyte differentiation and directly mediates macrophage susceptibility to infection by human immunodeficiency virus type 1.. J Virol.

[35] Zhu T, Mo H, Wang N, Nam DS, Cao Y (1993). Genotypic and phenotypic characterization of HIV-1 in patients with primary infection.. Science.

[36] van't Wout AB, Kootstra NA, Mulder-Kampinga GA, Albrecht-van Lent N, Scherpbier HJ (1994). Macrophage-tropic variants initiate human immunodeficiency virus type I infection after sexual, parenteral, and vertical transmission.. J Clin Invest.

[37] Keele BF, Giorgi EE, Salazar-Gonzalez JF, Decker JM, Pham KT (2008). Identification and characterization of transmitted and early founder virus envelopes in primary HIV-1 infection.. Proc Natl Acad Sci USA.

[38] Lee SH, Starkey PM, Gordon S (1985). Quantitative analysis of total macrophage content in adult mouse tissues. Immunochemical studies with monoclonal antibody F4/80.. J Exp Med.

[39] Peters PJ, Bhattacharya J, Hibbitts S, Dittmar MT, Simmons G (2004). Biological analysis of human immunodeficiency virus type 1 R5 envelopes amplified from brain and lymph node tissues of AIDS patients with neuropathology reveals two distinct tropism phenotypes and identifies envelopes in the brain that confer an enhanced tropism and fusigenicity for macrophages.. J Virol.

[40] Meng G, Wei X, Wu X, Sellers MT, Decker JM (2002). Primary intestinal epithelial cells selectively transfer R5 HIV-1 to CCR5^+^ cells.. Nat Med.

[41] Nabel G, Baltimore D (1987). An inducible transcription factor activates expression of human immunodeficiency virus in T cells.. Nature.

[42] Poli G, Kinter AL, Justement JS, Bressler P, Kehrl JH (1991). Transforming growth factor beta suppresses human immunodeficiency virus expression and replication in infected cells of the monocyte/macrophage lineage.. J Exp Med.

[43] Poli G, Kinter AL, Justement JS, Bressler P, Kehrl JH (1992). Retinoic acid mimics transforming growth factor beta in the regulation of human immunodeficiency virus expression in monocytic cells.. Proc Natl Acad Sci USA.

[44] Lazdins JK, Klimkait T, Woods-Cook K, Walker M, Alteri E (1991). *In vitro* effect of transforming growth factor-β on progression of HIV-1 infection in primary mononuclear phagocytes.. J Immunol.

[45] Griffin GE, Leung K, Folks TM, Kunkel S, Nabel GJ (1989). Activation of HIV gene expression during monocyte differentiation by induction of NF-κB.. Nature.

[46] Moses AV, Ibanez C, Gaynor R, Ghazal P, Nelson JA (1994). Differential role of long terminal repeat control elements for the regulation of basal and Tat-mediated transcription of the human immunodeficiency virus in stimulated and unstimulated primary human macrophages.. J Virol.

[47] Rittner K, Churcher MJ, Gait MJ, Karn J (1995). The human immunodeficiency virus long terminal repeat includes a specialised initiator element which is required for Tat-responsive transcription.. J Mol Biol.

[48] Li JM, Shen X, Hu PP, Wang XF (1998). Transforming growth factor β stimulates the human immunodeficiency virus 1 enhancer and requires NF-κB activity.. Mol Cell Biol.

[49] West MJ, Lowe AD, Karn J (2001). Activation of human immunodeficiency virus transcription in T cells revisited: NF-κB p65 stimulates transcriptional elongation.. J Virol.

[50] Dreikhausen U, Hiebenthal-Millow K, Bartels M, Resch K, Nourbakhsh M (2005). NF-κB-repressing factor inhibits elongation of human immunodeficiency virus type 1 transcription by DRB sensitivity-inducing factor.. Mol Cell Biol.

[51] Roulston A, Lin R, Beauparlant P, Wainberg MA, Hiscott J (1995). Regulation of human immunodeficiency virus type 1 and cytokine gene expression in myeloid cells by NF-κB/Rel transcription factors.. Microbiol Rev.

[52] DeLuca C, Petropoulos L, Zmeureanu D, Hiscott J (1999). Nuclear IκBβ maintains persistent NF-κB activation in HIV-1-infected myeloid cells.. J Biol Chem.

[53] Asin S, Taylor JA, Trushin S, Bren G, Paya CV (1999). Iκκ mediates NF-κB activation in human immunodeficiency virus-infected cells.. J Virol.

[54] Hiscott J, Kwon H, Genin P (2001). Hostile takeovers: viral appropriation of the NF-κB pathway.. J Clin Invest.

[55] Smith PD, Ochsenbauer-Jambor C, Smythies LE (2005). Intestinal macrophages: unique effector cells of the innate immune system.. Immunol Rev.

[56] Smith PD, Smythies LE, Shen R, Greenwell-Wild T, Gliozzi M (2011). Intestinal macrophages and response to microbial encroachment.. Mucosal Immunol.

[57] Smith PD, Fox CH, Masur H, Winter HS, Alling DW (1994). Quantitative analysis of mononuclear cells expressing human immunodeficiency virus type 1 RNA in esophageal mucosa.. J Exp Med.

[58] Maheshwari A, Smythies LE, Wu X, Novak L, Clements R (2006). Cytomegalovirus blocks intestinal stroma-induced down-regulation of macrophage HIV-1 infection.. J Leuk Biol.

[59] Huff KR, Akhtar LN, Fox AL, Cannon JA, Smith PD (2011). Extracellular matrix-associated cytokines regulate CD4^+^ effector T-cell responses in the human intestinal mucosa.. Mucosal Immunol epub.

[60] Salazar-Gonzalez JF, Salazar MG, Keele BF, Learn GH, Giorgi EE (2009). Genetic identity, biological phenotype, and evolutionary pathways of transmitted/founder viruses in acute and early HIV-1 infection.. J Exp Med.

[61] Li H, Bar KJ, Wang S, Decker JM, Chen Y (2010). High Multiplicity Infection by HIV-1 in Men Who Have Sex with Men.. PLoS Pathog.

[62] Peng G, Lei KJ, Jin W, Greenwell-Wild T, Wahl SM (2006). Induction of APOBEC3 family proteins, a defensive maneuver underlying interferon-induced anti-HIV-1 activity.. J Exp Med.

[63] Pion M, Granelli-Piperno A, Mangeat B, Stalder R, Correa R (2006). APOBEC3G/3F mediates intrinsic resistance of monocyte-derived dendritic cells to HIV-1 infection.. J Exp Med.

[64] Wang X, Ye L, Hou W, Zhou Y, Wang YJ (2009). Cellular microRNA expression correlates with susceptibility of monocytes/macrophages to HIV-1 infection.. Blood.

[65] Sung TL, Rice AP (2009). miR-198 inhibits HIV-1 gene expression and replication in monocytes and its mechanism of action appears to involve repression of cyclin T1.. PLoS Pathog.

[66] Cullen BR (2006). Is RNA interference involved in intrinsic antiviral immunity in mammals?. Nat Immunol.

[67] Swaminathan S, Zaunders J, Wilkinson J, Suzuki K, Kelleher AD (2009). Does the presence of anti-HIV miRNAs in monocytes explain their resistance to HIV-1 infection?. Blood.

[68] Kaushik R, Zhu X, Stranska R, Wu Y, Stevenson M (2009). A cellular restriction dictates the permissivity of nondividing monocytes/macrophages to lentivirus and gammaretrovirus infection.. Cell Host Microbe.

[69] Bergamaschi A, David A, Le Rouzic E, Nisole S, Barre-Sinoussi F (2009). The CDK inhibitor p21Cip1/WAF1 is induced by FcγR activation and restricts the replication of human immunodeficiency virus type 1 and related primate lentiviruses in human macrophages.. J Virol.

[70] Zhang J, Scadden DT, Crumpacker CS (2007). Primitive hematopoietic cells resist HIV-1 infection via p21.. J Clin Invest.

[71] Honda Y, Rogers L, Nakata K, Zhao BY, Pine R (1998). Type I interferon induces inhibitory 16-kD CCAAT/enhancer binding protein (C/EBP)β, repressing the HIV-1 long terminal repeat in macrophages: pulmonary tuberculosis alters C/EBP expression, enhancing HIV-1 replication.. J Exp Med.

[72] Hoshino Y, Nakata K, Hoshino S, Honda Y, Tse DB (2002). Maximal HIV-1 replication in alveolar macrophages during tuberculosis requires both lymphocyte contact and cytokines.. J Exp Med.

[73] Smith PD, Janoff EN, Mosteller-Barnum M, Merger M, Orenstein JM (1997). Isolation and purification of CD14-negative mucosal macrophages from normal human small intestine.. J Immunol Meth.

[74] Smythies LE, Wahl LM, Smith PD (2006). Isolation and purification of human intestinal macrophages.. Curr Protocol Immunol.

[75] Wahl LM, Wahl SM, Smythies LE, Smith PD (2006). Isolation of human monocyte populations.. Curr Protocol Immunol.

[76] Shen R, Smythies LE, Clements RH, Novak L, Smith PD (2010). Dendritic cells transmit HIV-1 through human small intestinal mucosa.. J Leukoc Biol.

[77] Shen R, Drelichman ER, Bimczok D, Ochsenbauer C, Kappes JC (2010). GP41-specific antibody blocks cell-free HIV type 1 transcytosis through human rectal mucosa and model colonic epithelium.. J Immunol.

[78] Wei X, Decker JM, Liu HM, Zhang Z, Arani RB (2002). Emergence of resistant human immunodeficiency virus type 1 in patients receiving fusion inhibitor (T-20) monotherapy.. Antimicrob Agents Chemother.

[79] Kutsch O, Benveniste EN, Shaw GM, Levy DN (2002). Direct and quantitative single-cell analysis of human immunodeficiency virus type 1 reactivation from latency.. J Virol.

